# (4aR*,8aS*)-2,3-Diphenyl-4a,5,6,7,8,8a-hexa­hydro­quinoxaline

**DOI:** 10.1107/S1600536812028061

**Published:** 2012-06-27

**Authors:** W. Chen, K.-S. Tang, L.-Y. Fan

**Affiliations:** a Department of Chemistry, Tongji University, Shanghai 200092, People’s Republic of China

## Abstract

In the title compound, C_20_H_20_N_2_, the quinoxaline ring adopts a very distorted half-chair conformation [N=C—C=N = 22.7 (2)° for the nominally coplanar atoms] and the cyclo­hexane ring adopts a chair conformation. The quinoxaline and cyclo­hexane rings are *cis*-fused. The two phenyl rings form a dihedral angle of 63.88 (7)°.

## Related literature

For background to dihydro­pyrazine derivatives, see: Raw *et al.* (2003 ▶). For related structures, see: Reich *et al.* (2004 ▶); Wang *et al.* (2008) ▶.
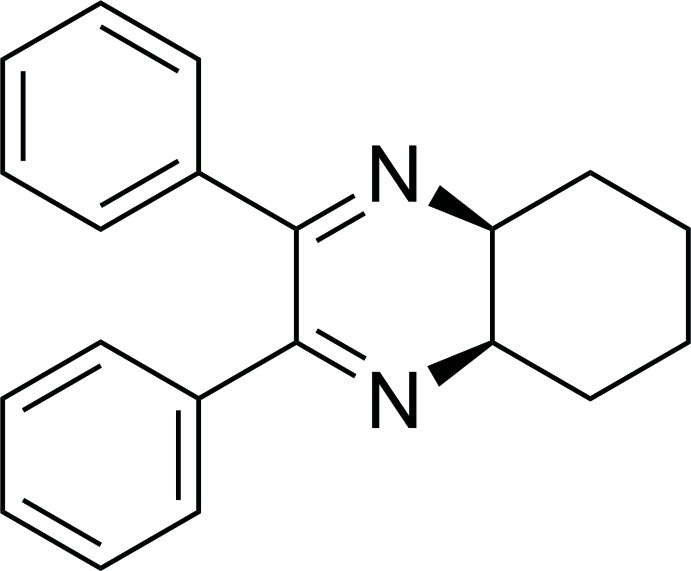


## Experimental

### 

#### Crystal data

C_20_H_20_N_2_
*M* *_r_* = 288.38
Orthorhombic, 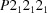

*a* = 6.3546 (1) Å
*b* = 13.4894 (2) Å
*c* = 19.1921 (3) Å
*V* = 1645.14 (4) Å^3^
*Z* = 4
Cu *K*α radiation
μ = 0.52 mm^−1^
*T* = 293 K
0.20 × 0.20 × 0.10 mm

#### Data collection

Bruker SMART APEX CCD diffractometer
Absorption correction: multi-scan (*SADABS*; Bruker, 2004) ▶ *T*_min_ = 0.902, *T*_max_ = 0.949
4074 measured reflections
2739 independent reflections
2703 reflections with *I* > 2σ(*I*)
*R*_int_ = 0.010

#### Refinement

*R*[*F* ^2^ > 2σ(*F* ^2^)] = 0.036
*wR*(*F* ^2^) = 0.099
*S* = 1.05
2739 reflections
199 parameters
H-atom parameters constrained
Δρ_max_ = 0.14 e Å^−3^
Δρ_min_ = −0.14 e Å^−3^

### 

Data collection: *APEX2* (Bruker, 2004) ▶; cell refinement: *SAINT-Plus* (Bruker, 2001) ▶; data reduction: *SAINT-Plus*; program(s) used to solve structure: *SHELXS97* (Sheldrick, 2008 ▶); program(s) used to refine structure: *SHELXL97* (Sheldrick, 2008 ▶); molecular graphics: *SHELXTL* (Sheldrick, 2008 ▶); software used to prepare material for publication: *SHELXL97*.

## Supplementary Material

Crystal structure: contains datablock(s) I, global. DOI: 10.1107/S1600536812028061/hb6855sup1.cif

Structure factors: contains datablock(s) I. DOI: 10.1107/S1600536812028061/hb6855Isup2.hkl

Supplementary material file. DOI: 10.1107/S1600536812028061/hb6855Isup3.cml

Additional supplementary materials:  crystallographic information; 3D view; checkCIF report

## Figures and Tables

**Fig. 1.**
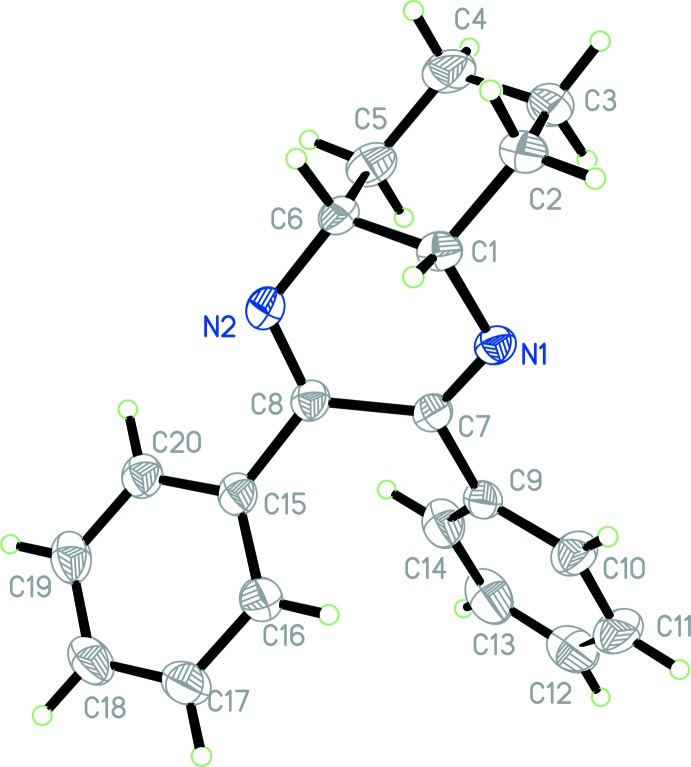
The molecular structure of the title compound with displacement ellipsoids drawn at the 30% probability level.

## supplementary crystallographic information

### Comment

Dihydropyrazine ring systems are found as a key unit in natural products and biochemical materials (Raw *et al.*, 2003). Therefore, the synthesis of dihydropyrazine derivatives attract much interest in organic chemistry. In this respect, we report herein the crystal structure of the title compound.

In the title compound, Fig. 1, the quinoxaline ring has a very distorted half-chair conformation and the cyclohexane ring has a chair conformation. The dihedral angle between the two benzene rings is 63.88 (7)°. The lengths of the C═N bonds [1.2678 (18)Å] are comparable to those in similar compounds (Wang *et al.*, 2008; Reich *et al.*, 2004).

### Experimental

2-hydroxy-1,2-diphenylethanone (0.212 g, 1 mmol) and YbCl_3_ (0.028 g, 0.1 mmol) were dissolved in 5 ml EtOH and stirred until the solid dissolved completely in refluxing, then (1*R*,2*S*)-cyclohexane-1,2-diamine (0.171 g, 1.5 mmol) and H_2_O_2_ (0.022 g, 0.2 mmol) were added into the mixture. After 30 min, TLC showed the reaction to be complete. The reaction mixture was cooled to room temperature. The solvent was evaporated in vacuum and the product purified by column chromatography on neutral alumina deactivated with H_2_O (6 wt%) (PE-EtOAc = 5:1) to give the title compound. The compound was recrystallized from methanol to give yellow blocks.

### Refinement

Hydrogen atoms were positioned geometrically and allowed to ride on their parent atoms, with C–H = 0.93–0.98 Å, and with *U*_iso_(H) values set to 1.2*U*_eq_(C).

### Crystal data

**Table:**

C_20_H_20_N_2_	*F*(000) = 616
*M**_r_* = 288.38	*D*_x_ = 1.164 Mg m^−^^3^
Orthorhombic, *P*2_1_2_1_2_1_	Cu *K*α radiation, λ = 1.54178 Å
Hall symbol: P 2ac 2ab	Cell parameters from 2779 reflections
*a* = 6.3546 (1) Å	θ = 3.3–72.4°
*b* = 13.4894 (2) Å	µ = 0.52 mm^−^^1^
*c* = 19.1921 (3) Å	*T* = 293 K
*V* = 1645.14 (4) Å^3^	Block, yellow
*Z* = 4	0.20 × 0.20 × 0.10 mm

### Data collection

**Table:**

Bruker SMART APEX CCD diffractometer	2739 independent reflections
Radiation source: fine-focus sealed tube	2703 reflections with *I* > 2σ(*I*)
Graphite monochromator	*R*_int_ = 0.010
phi and ω scans	θ_max_ = 72.6°, θ_min_ = 5.7°
Absorption correction: multi-scan (*SADABS*; Bruker, 2004)	*h* = −7→5
*T*_min_ = 0.902, *T*_max_ = 0.949	*k* = −15→16
4074 measured reflections	*l* = −17→23

### Refinement

**Table:**

Refinement on *F*^2^	Secondary atom site location: difference Fourier map
Least-squares matrix: full	Hydrogen site location: difference Fourier map
*R*[*F*^2^ > 2σ(*F*^2^)] = 0.036	H-atom parameters constrained
*wR*(*F*^2^) = 0.099	*w* = 1/[σ^2^(*F*_o_^2^) + (0.0617*P*)^2^ + 0.1364*P*] where *P* = (*F*_o_^2^ + 2*F*_c_^2^)/3
*S* = 1.05	(Δ/σ)_max_ < 0.001
2739 reflections	Δρ_max_ = 0.14 e Å^−^^3^
199 parameters	Δρ_min_ = −0.14 e Å^−^^3^
0 restraints	Absolute structure: unk
Primary atom site location: structure-invariant direct methods	

### Special details

**Table:**

Geometry. All e.s.d.'s (except the e.s.d. in the dihedral angle between two l.s. planes) are estimated using the full covariance matrix. The cell e.s.d.'s are taken into account individually in the estimation of e.s.d.'s in distances, angles and torsion angles; correlations between e.s.d.'s in cell parameters are only used when they are defined by crystal symmetry. An approximate (isotropic) treatment of cell e.s.d.'s is used for estimating e.s.d.'s involving l.s. planes.
Refinement. Refinement of *F*^2^ against ALL reflections. The weighted *R*-factor *wR* and goodness of fit *S* are based on *F*^2^, conventional *R*-factors *R* are based on *F*, with *F* set to zero for negative *F*^2^. The threshold expression of *F*^2^ > σ(*F*^2^) is used only for calculating *R*-factors(gt) *etc*. and is not relevant to the choice of reflections for refinement. *R*-factors based on *F*^2^ are statistically about twice as large as those based on *F*, and *R*- factors based on ALL data will be even larger.

### Fractional atomic coordinates and isotropic or equivalent isotropic displacement parameters (Å^2^)

**Table:**

	*x*	*y*	*z*	*U*_iso_*/*U*_eq_	
N1	0.18801 (18)	0.39372 (8)	0.62663 (6)	0.0410 (3)	
N2	−0.0579 (2)	0.33893 (9)	0.50829 (6)	0.0492 (3)	
C1	0.1165 (2)	0.29069 (10)	0.61739 (7)	0.0419 (3)	
H1B	0.2255	0.2542	0.5920	0.050*	
C2	0.0862 (3)	0.24167 (12)	0.68822 (8)	0.0544 (4)	
H2B	0.0632	0.1712	0.6818	0.065*	
H2C	0.2128	0.2501	0.7158	0.065*	
C3	−0.0991 (3)	0.28592 (15)	0.72680 (8)	0.0654 (5)	
H3A	−0.0700	0.3549	0.7372	0.079*	
H3B	−0.1183	0.2512	0.7706	0.079*	
C4	−0.2993 (3)	0.27893 (16)	0.68428 (11)	0.0674 (5)	
H4A	−0.4138	0.3100	0.7096	0.081*	
H4B	−0.3353	0.2098	0.6772	0.081*	
C5	−0.2729 (2)	0.32958 (14)	0.61387 (10)	0.0605 (4)	
H5A	−0.3992	0.3200	0.5863	0.073*	
H5B	−0.2536	0.4002	0.6208	0.073*	
C6	−0.0850 (2)	0.28777 (10)	0.57496 (7)	0.0453 (3)	
H6A	−0.1155	0.2181	0.5647	0.054*	
C7	0.1606 (2)	0.45124 (10)	0.57517 (7)	0.0388 (3)	
C8	0.0606 (2)	0.41514 (9)	0.50853 (7)	0.0414 (3)	
C9	0.2231 (3)	0.55791 (10)	0.58259 (7)	0.0442 (3)	
C10	0.4058 (3)	0.58177 (13)	0.61799 (9)	0.0591 (4)	
H10A	0.4885	0.5318	0.6371	0.071*	
C11	0.4658 (4)	0.68056 (16)	0.62502 (11)	0.0769 (6)	
H11A	0.5903	0.6965	0.6479	0.092*	
C12	0.3419 (5)	0.75417 (13)	0.59834 (10)	0.0843 (8)	
H12A	0.3823	0.8201	0.6031	0.101*	
C13	0.1589 (5)	0.73123 (13)	0.56464 (10)	0.0803 (7)	
H13A	0.0740	0.7816	0.5472	0.096*	
C14	0.0993 (4)	0.63297 (11)	0.55626 (9)	0.0614 (4)	
H14A	−0.0247	0.6177	0.5328	0.074*	
C15	0.1024 (3)	0.46329 (10)	0.43996 (7)	0.0444 (3)	
C16	0.2983 (3)	0.50275 (13)	0.42434 (8)	0.0535 (4)	
H16A	0.4023	0.5054	0.4584	0.064*	
C17	0.3394 (3)	0.53842 (14)	0.35770 (9)	0.0631 (4)	
H17A	0.4713	0.5644	0.3473	0.076*	
C18	0.1862 (4)	0.53548 (13)	0.30698 (9)	0.0634 (5)	
H18A	0.2147	0.5590	0.2624	0.076*	
C19	−0.0101 (3)	0.49741 (13)	0.32258 (8)	0.0605 (4)	
H19A	−0.1142	0.4958	0.2886	0.073*	
C20	−0.0522 (3)	0.46158 (11)	0.38877 (8)	0.0514 (4)	
H20A	−0.1848	0.4362	0.3990	0.062*	

### Atomic displacement parameters (Å^2^)

**Table:**

	*U*^11^	*U*^22^	*U*^33^	*U*^12^	*U*^13^	*U*^23^
N1	0.0362 (5)	0.0423 (6)	0.0445 (6)	−0.0035 (5)	0.0028 (5)	−0.0021 (5)
N2	0.0577 (7)	0.0453 (6)	0.0446 (6)	−0.0099 (6)	−0.0039 (6)	−0.0055 (5)
C1	0.0413 (7)	0.0367 (6)	0.0476 (7)	0.0000 (6)	0.0081 (6)	−0.0014 (6)
C2	0.0568 (9)	0.0541 (8)	0.0523 (8)	−0.0089 (7)	0.0006 (7)	0.0092 (7)
C3	0.0728 (11)	0.0777 (11)	0.0458 (8)	−0.0250 (10)	0.0162 (8)	−0.0067 (8)
C4	0.0529 (9)	0.0777 (11)	0.0716 (10)	−0.0188 (9)	0.0248 (8)	−0.0114 (10)
C5	0.0380 (7)	0.0664 (10)	0.0771 (11)	−0.0071 (7)	0.0026 (7)	−0.0018 (9)
C6	0.0510 (7)	0.0387 (6)	0.0462 (7)	−0.0117 (6)	0.0013 (6)	−0.0057 (6)
C7	0.0368 (6)	0.0378 (6)	0.0419 (6)	−0.0007 (5)	0.0043 (5)	−0.0047 (5)
C8	0.0455 (7)	0.0360 (6)	0.0427 (6)	0.0001 (6)	0.0007 (6)	−0.0060 (5)
C9	0.0560 (8)	0.0390 (7)	0.0376 (6)	−0.0067 (6)	0.0075 (6)	−0.0065 (5)
C10	0.0606 (9)	0.0534 (8)	0.0632 (9)	−0.0126 (8)	0.0026 (8)	−0.0113 (7)
C11	0.0866 (13)	0.0702 (12)	0.0738 (12)	−0.0356 (11)	0.0105 (11)	−0.0232 (10)
C12	0.148 (2)	0.0435 (9)	0.0617 (10)	−0.0306 (12)	0.0243 (14)	−0.0141 (8)
C13	0.142 (2)	0.0406 (8)	0.0581 (9)	0.0044 (11)	0.0039 (13)	−0.0052 (7)
C14	0.0894 (12)	0.0426 (7)	0.0522 (8)	0.0040 (8)	−0.0030 (9)	−0.0040 (6)
C15	0.0567 (8)	0.0340 (6)	0.0425 (7)	0.0010 (6)	0.0005 (6)	−0.0050 (5)
C16	0.0607 (9)	0.0529 (8)	0.0469 (7)	−0.0056 (8)	0.0019 (7)	−0.0016 (7)
C17	0.0737 (11)	0.0616 (9)	0.0539 (8)	−0.0074 (9)	0.0126 (8)	0.0062 (7)
C18	0.0919 (13)	0.0539 (8)	0.0443 (8)	0.0018 (9)	0.0072 (8)	0.0071 (7)
C19	0.0833 (12)	0.0513 (8)	0.0469 (8)	0.0057 (9)	−0.0121 (8)	0.0012 (7)
C20	0.0616 (9)	0.0428 (7)	0.0496 (8)	−0.0009 (7)	−0.0029 (7)	−0.0028 (6)

### Geometric parameters (Å, º)

**Table:**

N1—C7	1.2680 (18)	C9—C14	1.378 (2)
N1—C1	1.4728 (17)	C9—C10	1.383 (2)
N2—C8	1.2745 (19)	C10—C11	1.393 (3)
N2—C6	1.4638 (19)	C10—H10A	0.9300
C1—C6	1.518 (2)	C11—C12	1.367 (4)
C1—C2	1.524 (2)	C11—H11A	0.9300
C1—H1B	0.9800	C12—C13	1.366 (4)
C2—C3	1.513 (2)	C12—H12A	0.9300
C2—H2B	0.9700	C13—C14	1.388 (3)
C2—H2C	0.9700	C13—H13A	0.9300
C3—C4	1.515 (3)	C14—H14A	0.9300
C3—H3A	0.9700	C15—C16	1.387 (2)
C3—H3B	0.9700	C15—C20	1.390 (2)
C4—C5	1.523 (3)	C16—C17	1.391 (2)
C4—H4A	0.9700	C16—H16A	0.9300
C4—H4B	0.9700	C17—C18	1.377 (3)
C5—C6	1.517 (2)	C17—H17A	0.9300
C5—H5A	0.9700	C18—C19	1.381 (3)
C5—H5B	0.9700	C18—H18A	0.9300
C6—H6A	0.9800	C19—C20	1.385 (2)
C7—C9	1.4996 (18)	C19—H19A	0.9300
C7—C8	1.5087 (18)	C20—H20A	0.9300
C8—C15	1.4914 (19)		
			
C7—N1—C1	116.18 (11)	C9—C7—C8	120.12 (12)
C8—N2—C6	116.52 (12)	N2—C8—C15	116.95 (12)
N1—C1—C6	110.45 (11)	N2—C8—C7	120.82 (12)
N1—C1—C2	109.94 (12)	C15—C8—C7	122.18 (11)
C6—C1—C2	111.15 (12)	C14—C9—C10	119.24 (15)
N1—C1—H1B	108.4	C14—C9—C7	121.25 (14)
C6—C1—H1B	108.4	C10—C9—C7	119.49 (14)
C2—C1—H1B	108.4	C9—C10—C11	120.01 (19)
C3—C2—C1	111.32 (14)	C9—C10—H10A	120.0
C3—C2—H2B	109.4	C11—C10—H10A	120.0
C1—C2—H2B	109.4	C12—C11—C10	120.1 (2)
C3—C2—H2C	109.4	C12—C11—H11A	120.0
C1—C2—H2C	109.4	C10—C11—H11A	120.0
H2B—C2—H2C	108.0	C13—C12—C11	120.20 (17)
C2—C3—C4	111.43 (13)	C13—C12—H12A	119.9
C2—C3—H3A	109.3	C11—C12—H12A	119.9
C4—C3—H3A	109.3	C12—C13—C14	120.2 (2)
C2—C3—H3B	109.3	C12—C13—H13A	119.9
C4—C3—H3B	109.3	C14—C13—H13A	119.9
H3A—C3—H3B	108.0	C9—C14—C13	120.2 (2)
C3—C4—C5	110.93 (13)	C9—C14—H14A	119.9
C3—C4—H4A	109.5	C13—C14—H14A	119.9
C5—C4—H4A	109.5	C16—C15—C20	119.22 (14)
C3—C4—H4B	109.5	C16—C15—C8	121.16 (14)
C5—C4—H4B	109.5	C20—C15—C8	119.41 (14)
H4A—C4—H4B	108.0	C15—C16—C17	119.99 (16)
C6—C5—C4	110.90 (15)	C15—C16—H16A	120.0
C6—C5—H5A	109.5	C17—C16—H16A	120.0
C4—C5—H5A	109.5	C18—C17—C16	120.46 (18)
C6—C5—H5B	109.5	C18—C17—H17A	119.8
C4—C5—H5B	109.5	C16—C17—H17A	119.8
H5A—C5—H5B	108.0	C17—C18—C19	119.72 (15)
N2—C6—C5	110.33 (13)	C17—C18—H18A	120.1
N2—C6—C1	110.95 (11)	C19—C18—H18A	120.1
C5—C6—C1	112.96 (12)	C18—C19—C20	120.20 (17)
N2—C6—H6A	107.4	C18—C19—H19A	119.9
C5—C6—H6A	107.4	C20—C19—H19A	119.9
C1—C6—H6A	107.4	C19—C20—C15	120.39 (17)
N1—C7—C9	118.48 (12)	C19—C20—H20A	119.8
N1—C7—C8	121.37 (12)	C15—C20—H20A	119.8
